# Serotonin tranporter methylation and response to cognitive behaviour therapy in children with anxiety disorders

**DOI:** 10.1038/tp.2014.83

**Published:** 2014-09-16

**Authors:** S Roberts, K J Lester, J L Hudson, R M Rapee, C Creswell, P J Cooper, K J Thirlwall, J R I Coleman, G Breen, C C Y Wong, T C Eley

**Affiliations:** 1 MRC Social, Genetic and Developmental Psychiatry Centre, Institute of Psychiatry, King's College London, London UK; 2 Centre for Emotional Health, Department of Psychology, Macquarie University, Sydney, Australia; 3 Winnicott Research Unit, School of Psychology and Clinical Language Sciences, University of Reading, Reading, UK; 4 Department of Psychology, Stellenbosch University, Western Cape, South Africa

## Abstract

Anxiety disorders that are the most commonly occurring psychiatric disorders in childhood, are associated with a range of social and educational impairments and often continue into adulthood. Cognitive behaviour therapy (CBT) is an effective treatment option for the majority of cases, although up to 35–45% of children do not achieve remission. Recent research suggests that some genetic variants may be associated with a more beneficial response to psychological therapy. Epigenetic mechanisms such as DNA methylation work at the interface between genetic and environmental influences. Furthermore, epigenetic alterations at the serotonin transporter (*SERT*) promoter region have been associated with environmental influences such as stressful life experiences. In this study, we measured DNA methylation upstream of *SERT* in 116 children with an anxiety disorder, before and after receiving CBT. Change during treatment in percentage DNA methylation was significantly different in treatment responders vs nonresponders. This effect was driven by one CpG site in particular, at which responders increased in methylation, whereas nonresponders showed a decrease in DNA methylation. This is the first study to demonstrate differences in *SERT* methylation change in association with response to a purely psychological therapy. These findings confirm that biological changes occur alongside changes in symptomatology following a psychological therapy such as CBT.

## Introduction

Anxiety disorders have negative implications for many aspects of everyday functioning.^1,2^ They often arise during childhood and are not only associated with adult anxiety disorders but also with other forms of adult psychopathology such as depression.^3,4^ Cognitive behaviour therapy (CBT) is an effective treatment option for child and adolescent anxiety disorders with a remission rate of 56% following treatment, rising to around 65% at follow-up.^5,6^ Research exploring predictors of response to CBT in childhood anxiety disorders is somewhat mixed, but provides modest support for factors such as pretreatment symptom severity, comorbidity and parental psychopathology.^7, 8, 9, 10^ These factors are consistent with a possible genetic basis to CBT response.

Multiple genetic markers have been investigated for association with anxiety disorders and depression. Due to the success of anxiolytic and antidepressant drugs that target the serotonin transporter system^11,12^ and the critical role of the serotonin transporter in the termination of serotonergic neurotransmission, polymorphic variants of the serotonin transporter gene (*SERT)* have been widely studied. One of the best characterized of these variants is the serotonin transporter-linked polymorphic region, 5HTTLPR, which consists of a 43/44 bp insertion/deletion upstream of the transcription start site.^13,14^ Meta-analyses suggest that the short allelic variant of this region is associated with higher levels of anxiety and other related traits.^15^ This association is also subject to environmental influence, with studies of gene × environment interactions showing that the short allele carriers have the poorest outcomes (the highest levels of anxiety and depression) in high-stress, negative environments.^16,17^ However, individuals homozygous for the short allele also display better outcomes in low stress environments,^18, 19, 20, 21, 22^ suggesting that 5HTTLPR may represent an example of ‘Differential Susceptibility', or a higher sensitivity to environmental influences.^23^

Therapies such as CBT provide an excellent setting in which to explore environmental influences in a gene × environment interaction context as the timing of treatment is known, allowing for prospective assessment of the association between symptoms and genetic factors.^24^ Previously, we published data showing a significant association between 5HTTLPR genotype and response to CBT in children with anxiety disorders.^25^ At follow-up, compared with L allele carriers, children homozygous for the short allele were 20% more likely to be free of their primary anxiety disorder diagnosis, and 18.8% more likely to be free of all anxiety disorder diagnoses.^25^ Children homozygous for the short allele also showed a significantly greater reduction in symptom severity than those with one or more long alleles. These findings reflect an interaction between genetic (5HTTLPR) and environmental (CBT) influences on outcome.

Epigenetic mechanisms such as DNA methylation are thought to work at the interface between genes and the environment as they are sensitive to changes in environmental stimuli. The *SERT* promoter region has been shown to be particularly variable in DNA methylation across time,^26^ and differential DNA methylation patterns at this region have been associated with a range of negative life experiences. A study of *SERT* DNA methylation in adoptees found altered methylation patterns in those who experienced a range of negative life events and stressful situations such as child abuse and loss.^27, 28, 29^ In the case of unresolved loss or trauma, changes in DNA methylation associated with outcome were dependent on 5HTTLPR genotype. Monozygotic twins offer the opportunity to study the effects of environmental influences on *SERT* methylation while controlling for genetics. For example, in a study of monozygotic twins discordant for bullying victimization, increased *SERT* methylation patterns were identified in the bullied twins compared with their co-twins with no history of bullying.^30^

The investigation of DNA methylation and outcome following psychological therapy is a new area of research. One previous study of participants with borderline personality disorders (*n*=115) suggested an association between poorer response to intensive dialectical behaviour therapy and increases in DNA methylation of the brain-derived neurotrophic factor gene.^31^ Another study of veterans with posttraumatic stress disorder (*n*=16) found that higher pretreatment glucocorticoid-related *NR3C1* methylation predicted treatment outcome, whereas *FKBP5* gene methylation decreased across treatment time in association with recovery.^32^ However, some participants were also medicated in both studies.

In this study, we compared *SERT* DNA methylation change from pre- to post-treatment in 116 children receiving CBT for an anxiety disorder. Specifically, we compared mean changes in methylation during the treatment period for those who had and had not remitted from their primary anxiety disorder by the end of treatment and by the follow-up time point (6 months after the end of treatment). To our knowledge, this is the first study of response to a purely psychological therapy and changes in DNA methylation to focus on the *SERT* promoter region. Given the role played by DNA methylation in mediating the effect of the environment on emotional and behavioural outcomes, and previous findings that 5HTTLPR genotype is associated with treatment response, we hypothesized that treatment outcome would be associated with *SERT* DNA methylation changes during treatment.

## Materials and Methods

### Participants

#### Sample

Participants (*n*=116) represent a subsample of a larger longitudinal study exploring genetic predictors of therapy response. Subjects were recruited at clinical sites in Sydney, Australia (*n*=88) and Reading, UK (*n*=28), and provided buccal swab DNA samples at assessment sessions pre- and post-treatment, within 2 weeks of the first and last treatment session, respectively. Participants ranged in age from 6 to 13 years (mean age=9.34 years); 51.7% were male. All children completed a full course of manual-based CBT for a primary diagnosis of an anxiety disorder. Details of the specific treatment protocols at each site have been reported elsewhere.^25^ All trials and collection of samples were approved by Human Ethics and Biosafety Committees at both clinical sites. Parents provided informed consent, children provided assent. The storage and analysis of DNA for this study were approved by the Psychiatry, Nursing and Midwifery Research Ethics Sub-Committee at King's College London, London, UK.

#### Anxiety disorder diagnoses

Participants were diagnosed according to the Anxiety Disorders Interview Schedule for DSM-IV, Child and Parent Versions (ADIS-IV-C/P),^33^ and severity ratings (0–8) were made by graduate or clinical psychologists on the basis of composite parent and child reports. Anxiety disorder diagnoses corresponded to a clinical severity rating of 4 or greater. Clinical diagnoses were made at assessment sessions pre- and post-treatment, and also at a follow-up time point (6 months after the conclusion of treatment). Details of sample characteristics and primary anxiety disorder diagnoses made can be found in Table 1.

### Methods

#### DNA preparation

DNA was collected at pre- and post-treatment using buccal swabs, and extracted using established procedures designed to maximize the purity and yield of DNA from the sample.^34^ Extracted genomic DNA (510 ng) was treated with sodium bisulphite using the EZ-96 DNA Methylation Kit (Zymo Research, Orange, CA, USA).

#### Bisulphite PCR

The *SERT* region targeted in this study is situated upstream of the promoter. To aid amplification, the sequence was split into two smaller, overlapping amplicons of 200 and 296 bp that covered the whole region (NCBI build 37, chromosome 17; (1) 28562753–28563048, (2) 28563022–28563221, see Supplementary Information, Figure 1a). Bisulphite-PCR primers for each amplicon were designed using the Sequenom EpiDesigner software. PCR amplification was conducted using 45 cycles at an annealing temperature of 56 °C, with Qiagen Hot Star Taq DNA Polymerase (Qiagen, UK) and Sequenom MassCLEAVE tagged primers (Primer sequences in Supplementary Information Table 1, amplicon sequences in Supplementary Information Figure 1b). Each PCR was performed in duplicate for both amplicons per sample, and the PCR products pooled by amplicon to minimize PCR bias.

#### Sequenom EpiTYPER

DNA methylation was quantitatively measured using the Sequenom EpiTYPER system (Sequenom, San Diego, CA, USA), which has been described previously.^35^ The sample reported (*n*=116) refers to samples that had bisulphite-PCR product available for both pre- and posttreatment time points.

#### Quality control

Artificially fully methylated and unmethylated samples were included as assay controls. Probes detecting an average methylation of <5% were excluded from any analyses. Following stringent quality control, quantitative methylation data from six CpG sites was obtained.

#### Analyses

We considered two measures of treatment response. First was primary anxiety response, defined as the absence of the primary anxiety diagnosis. Second was a broad anxiety response, defined as the absence of all anxiety diagnoses. Clinical outcome was assessed at posttreatment and at follow-up (6 months after treatment). Average DNA methylation was calculated where data were available for at least five probes at both time points. Change in DNA methylation during treatment (from pre- to posttreatment) was calculated for all CpG sites, and also as an average across the region. All analyses were performed in SPSS (version 20.0, IBM Corp, Armonk, NY, USA).

To correct for multiple testing, a revised significance threshold was calculated using Matrix Spectral Decomposition.^36^ This method uses correlation between methylation levels at the CpG sites to give an effective number of independent variables to correct for. In our data, the estimated number of independent variables was 5.641, corresponding with a significant threshold of *P*=0.009.

## Results

Clinical outcome was in line with previous estimates. For primary and all anxiety diagnoses, remission rates were 48.3 and 29.3% at posttreatment, and 70.7 and 51.7% at follow-up (Table 2).

Percentage DNA methylation at pre- and posttreatment was not significantly different in the whole group at any CpG sites (Supplementary Table 2). However, there were significant differences between responders and nonresponders.

**Figure 1:**
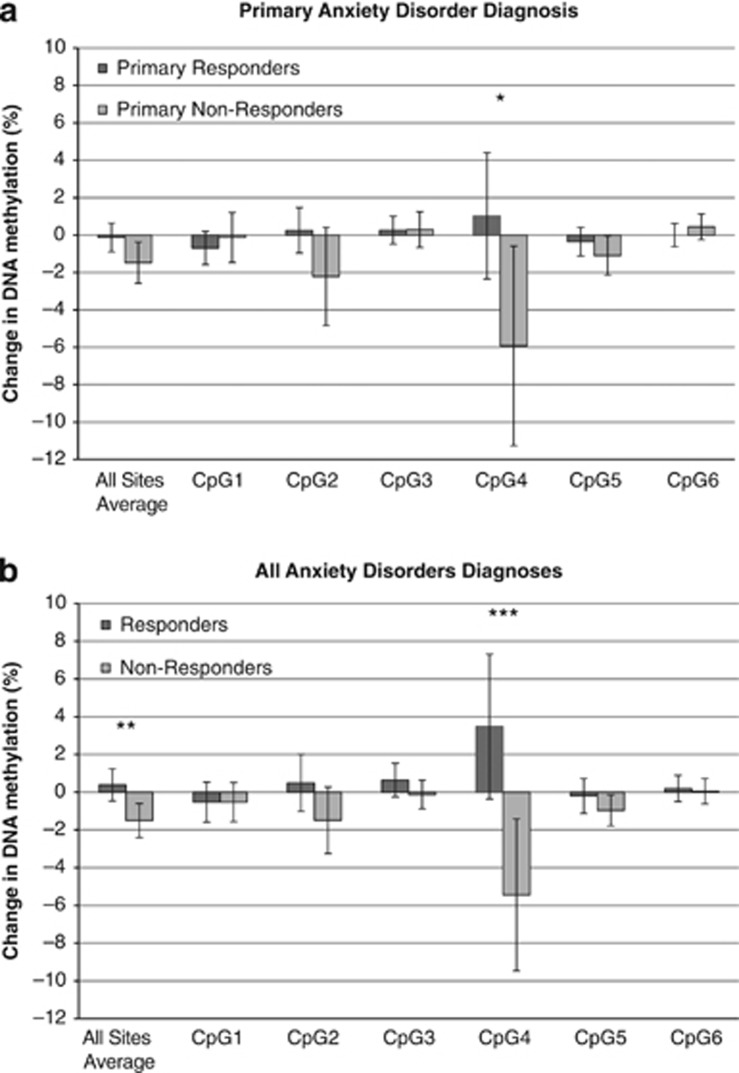
Change in DNA methylation from pre- to posttreatment in treatment repsonders and nonresponders by CpG site. (**a**) Primary anxiety responders, *P<0.05. (**b**) All anxiety responders **P=0.004, ***P=0.002.

For primary anxiety response, those defined as responders at follow-up showed a small increase in *SERT* methylation during the treatment period (from pre- to posttreatment, +1.1%) at CpG site 4, whereas nonresponders showed a larger decrease in methylation (nominally significant;−5.9%, *t*(91)=2.114, *P*=0.037, *d*=0.499; see Figure 1a). The difference in DNA methylation change as an average across the whole region was not statistically significant between responders and nonresponders (*t*(114)=1.904, *P*=0.059, *d*=0.395). When split by primary anxiety response at posttreatment, responders and nonresponders did not differ significantly in change in methylation (all sites average: *t*(107)=−0.363, *P*=0.717, *d*=−0.070. CpG site 4: *t*(87)=0.021, *P*=0.984, *d*=0.000). Finally, when looking at change in symptom severity for the primary anxiety disorder from posttreatment to follow-up, there was a significant difference in average DNA methylation change (during treatment) across the region between participants who continued to improve from posttreatment to follow-up and those who showed no improvement or worsened (*t*(95.45)=3.067, *P*=0.003, *d*=0.591).

For all anxiety response, there was a significant difference between treatment responders and nonresponders at follow-up in average DNA methylation change during treatment (Figure 1: *t*(114)=2.981, *P*=0.004, *d*=0.554). This effect was driven by CpG site 4 in particular (Figure 1: *t*(91)=3.146, *P*=0.002, *d*=0.65). At this CpG site, responders showed an increase in methylation during treatment (mean change=+3.48%), whereas nonresponders showed a decrease in methylation during treatment (mean change=−5.44%). (The same pattern of results for all tests was detected when including an additional participant on a stable dose of anxiety medication (reported in Supplementary Information).)

Of note, when split by response at posttreatment, all anxiety responders and nonresponders did not differ significantly in change in methylation (all sites average: *t*(110)=−0.700, *P*=0.486, *d*=−0.089. CpG site 4: *t*(87)=−0.400, *P*=0.690, *d*=0.140).

There were no significant differences in DNA methylation change at CpG site 4 or average methylation change across the region between participants split by age (older vs younger), gender or treatment site (test statistics; see Supplementary Table 3). Treatment response groups (responders vs nonresponders for both primary anxiety outcome and all anxiety outcome, and improvers vs those who did not during the follow-up period for primary anxiety outcome) did not differ on any demographic variables tested (Supplementary Table 3).

## Discussion

Our findings suggest that treatment responders and nonresponders have differential patterns of methylation change across a course of CBT. Responders showed an increase in percentage DNA methylation during treatment at one CpG site in the *SERT* promoter region, whereas nonresponders decreased in percentage DNA methylation. The results of the current study are comparable to those reported by a previous study of response to psychological therapy in borderline personality disorder, where responders and nonresponders showed a difference in the direction of change in brain-derived neurotrophic factor DNA methylation.^31^

Interestingly, this effect was not observable when participants were split by treatment outcome at posttreatment. However, there was a significant difference in average DNA methylation change across the region between those who continued to improve during the follow-up period, and those who worsened. The period between posttreatment and follow-up typically represents a period of consolidation, where the child continues to practice the skills and techniques they have learnt and developed during treatment. These results are in line with previous research from our team that found no effect of genotype immediately posttreatment, but that individuals homozygous for the S allele of the 5HTTLPR were 20% more likely to respond to CBT at follow-up.^25^

In this study, significant differences in DNA methylation change during treatment were found for those free of all anxiety disorder diagnoses at follow-up, but not for those only free of their primary anxiety diagnosis. Effect sizes using the all anxiety response measures were medium, but were small to medium for primary anxiety response.^37^ These findings suggest that the association between DNA methylation change and treatment response is strongest for those who are more general responders, rather than those whose response is disorder specific.

Psychological therapies such as CBT involve exposing the individual to the feared stimuli, and helping them to change the thought processes that surround fear and anxiety-provoking situations. Neuroimaging studies of CBT in anxiety disorders have demonstrated that there are functional changes in brain activation patterns across the course of treatment, and furthermore that these patterns are associated with differential response to CBT.^38, 39, 40, 41, 42, 43, 44^ The findings that changes in neural activation patterns are associated with psychological outcomes, such as fear responses and remission, provide evidence that CBT is associated with biological processes. Studies in both humans and macaques have implied that epigenetic regulation of the *SERT* promoter region is associated with levels of SERT gene expression and differences in behaviour following environmental stress.^28,45, 46, 47^ Higher DNA methylation is often (though not always) associated with reduced gene expression.^48,49^ In this study, we find that anxious children who respond to CBT have increased levels of *SERT* DNA methylation over time. It is possible that this increase in methylation may correspond with a reduction in expression. Reduced gene expression is commonly linked with the short allele of the 5HTTLPR genotype,^13^ which we have previously shown to be associated with response to CBT.^25^ The demonstration that *SERT* DNA methylation patterns are associated with differential outcomes following CBT suggests that this may be a plausible mechanism by which the biological changes associated with alterations in thought processes occur.

The results should be considered preliminary as they have not yet been replicated. This study also has some limitations. First, the sample is relatively small, and was too small to look at the effect of genotype. However, we note that the power of the study is enhanced by the collection of samples from multiple time points. Second, we have no DNA samples to measure percentage DNA methylation at the follow-up time point. Given the findings from this and earlier studies that response to treatment at follow-up is associated with both 5HTTLPR genotype and changes in DNA methylation, future studies investigating potential biological mechanisms of response would benefit from including DNA samples at follow-up. Third, the DNA used was derived from buccal swabs. The design of this study meant that it was necessary to utilize peripheral tissue samples as they were collected from living participants at multiple time points. Furthermore, the use of a child sample precludes other tissues types. However, although tissue-specific differences in DNA methylation have been previously documented,^50^ recent research suggests that buccal swabs may be more representative than other peripheral tissues such as blood, as they have less cell heterogeneity and originate from the same developmental pathway as brain tissue.^51^ Finally, we were not able to measure *SERT* expression in this sample, and were therefore unable to investigate the functionality of the observed changes in DNA methylation at CpG site 4. However, differential DNA methylation in the *SERT* promoter region has previously been shown to be associated with altered gene expression.^28^

In conclusion, we have demonstrated that DNA methylation change during treatment is associated with response to CBT in children with anxiety disorders. The results, which require replication, support the hypothesis that response to environmental influences such as psychological therapy is associated with changes at a biological level.

## Figures and Tables

**Table 1:** Sample characteristics

*Sample characteristics*	
*Clinical site*	
Sydney	*n*=88 (75.9%)
Reading	*n*=28 (24.1%)
	
*Age*	
Range (mean)	6–13 (9.34)
	
*Primary anxiety diagnosis*	
Generalized anxiety disorder	*n*=56 (48.3%)
Specific phobia	*n*=20 (17.2%)
Separation anxiety disorder	*n*=16 (13.8%)
Social anxiety disorder	*n*=14 (12.1%)
Obsessive compulsive disorder	*n*=9 (7.8%)
Panic disorder/agoraphobia	*n*=1 (0.9%)
	
*Gender*	
Male	*n*=60 (51.7%)
Female	*n*=56 (48.3%)
	
*Treatment group*	
Individual CBT	*n*=95 (81.9%)
Group CBT	*n*=9 (7.8%)
Guided self help (e.g., bibliotherapy with therapist support)	*n*=12 (10.3%)

Abbreviation: CBT, cognitive behaviour therapy.

**Table 2:** Treatment outcome

*Treatment outcome*—*free of diagnosis*	
*Posttreatment*	
Primary anxiety disorder	*n*=56 (48.3%)
All anxiety disorder diagnoses	*n*=34 (29.3%)
	
*Follow-up*	
Primary anxiety disorder	*n*=82 (70.7%)
All anxiety disorder diagnoses	*n*=60 (51.7%)

## Notes

RMR and JLH are authors of the Cool Kids program. CC was the joint author of book used in treatment within Overcoming trial and receives royalties from sales of the book. The remaining authors declare no conflict of interest.
