# Supplementary material for: Serotonin tranporter methylation and response to cognitive behaviour therapy in children with anxiety disorders
Source: Transl Psychiatry. 2014 Sep 16;4(9):e444–. doi: 10.1038/tp.2014.83 (PMC4203012; doi:10.1038/tp.2014.83)
Supplement: Supplementary Information [file tp201483x1.doc]

**Supplementary Information**

Table 1. Primer sequences

| Amplicon | Length | Primer sequence (tags in lower case) | |
| --- | --- | --- | --- |
| 1 | 200bp | F: | aggaagagagTTGTTAGGTTTTAGGAAGAAAGAGAGA |
|  |  | R: | cagtaatacgactcactatagggagaaggctAAAAATCCTAACTTTCCTACTCTTTAACT |
| 2 | 296bp | F: | aggaagagagTTAAAGAGTAGGAAAGTTAGGATTTTT |
|  |  | R: | cagtaatacgactcactatagggagaaggctAACCCTCACATAATCTAATCTCTAAAT |

Table 2. No significant differences in methylation; No effect of time on methylation - no CpG sites are statistically different at pre- and post-treatment. No effect of age, gender or treatment site on change in methylation from pre- to post-treatment.

| Condition | | DNA methylation (%) | | Test statistics | | |
| --- | --- | --- | --- | --- | --- | --- |
| Change from Pre- to Post-Treatment | CpG Site | Pre  Mean (Range) | Post  Mean (Range) | t | df | Sig (p) |
|  | Average | 8.8 (4-19) | 8.3 (5-20) | 1.60 | 115 | .11 |
|  | 1 | 8.0 (2-16) | 7.5 (0-15) | 1.40 | 109 | .17 |
|  | 2 | 10.9 (2-40) | 10.4 (3-25) | .78 | 109 | .44 |
|  | 3 | 6.7 (0-13) | 6.9 (0-14) | -.90 | 114 | .37 |
|  | 4 | 10.8 (0-50) | 9.9 (0-71) | .57 | 92 | .57 |
|  | 5 | 9.9 (4-25) | 9.3 (5-15) | 1.80 | 115 | .08 |
|  | 6 | 5.3 (0-17) | 5.4 (0-11) | -.53 | 115 | .60 |
| Variable | | Mean DNA methylation change | | Test statistics | | |
| Age | CpG Site | Younger (6-9yo)  n = 61 | Older (10-13yo)  n = 55 | t | df | Sig (p) |
|  | Average | -.8 | -.2 | .81 | 114 | .42 |
|  | 4 | -2.7 | 1.3 | 1.35 | 91 | .18 |
| Gender | CpG Site | Male  n = 60 | Female  n = 56 | t | df | Sig (p) |
|  | Average | -.9 | -.1 | -1.12 | 114 | .27 |
|  | 4 | -.7 | -1.0 | .11 | 91 | .91 |
| Treatment Site | CpG Site | Sydney  n = 88 | Reading  n = 28 | t | df | Sig (p) |
|  | Average | -.4 | -.9 | -.62 | 114 | .54 |
|  | 4 | -.8 | -.8 | .01 | 91 | .99 |
| Ethnicity | CpG Site | White  n = 76 | Not White  n = 23 | t | df | Sig (p) |
|  | Average | -.8 | .3 | 97 | 97 | .239 |
|  | 4 | -2.4 | 3.9 | 1.44 | 77 | .154 |

| Outcome measure | Clinical Variable | | | Test statistics | | |
| --- | --- | --- | --- | --- | --- | --- |
| Presence vs Absence of All Anxiety Disorders | |  | **χ^2^** | | df | p |
|  |  | Age | 7.79 | | 7 | 0.35 |
|  |  | Gender | 0.53 | | 1 | 0.58 |
|  |  | Site | 0.04 | | 1 | 1.00 |
|  |  | Ethnicity | 0.19 | | 1 | 0.81 |
| Presence vs Absence of Primary Anxiety Disorder | |  | **χ^2^** | | df | p |
|  |  | Age | 10.82 | | 7 | 0.15 |
|  |  | Gender | 0.06 | | 1 | 0.84 |
|  |  | Pre-treatment Severity | 5.40 | | 4 | 0.25 |
|  |  | Site | 0.01 | | 1 | 1.00 |
|  |  | Ethnicity | 0.15 | | 1 | 0.80 |
| Improvement vs No Improvement/Worsened for Primary Anxiety Disorder Symptom Severity | |  | **χ^2^** | | df | p |
|  |  | Age | 8.76 | | 7 | 0.27 |
|  |  | Gender | 0.22 | | 1 | 0.70 |
|  |  | Pre-treatment Severity | 2.21 | | 4 | 0.70 |
|  |  | Site | 0.01 | | 1 | 1.00 |
|  |  | Ethnicity | 0.01 | | 1 | 1.00 |
|  | | | | | | |

Table 3. No significant differences between response groups (primary and all anxiety disorders) for demographic information.

*Results including individual on anxiety medication*

There was a significant difference between responders (free of all anxiety disorder diagnoses at follow-up) and non-responders in change in DNA methylation from pre- to post-treatment across the region (*t*(115) =3.116, *p*=.002). This result appears to be driven by CpG site 4 in particular, where responders showed an increase in methylation (+3.5%) and non-responders showed a decreased in methylation (-6.1%, *t*(92) = 3.326, *p* = .001).

**Supplementary Figures**: Genomic location (adapted from UCSC NCBI36/h18 Genome Browser) and amplified sequences of the *SERT* DNA methylation assays included in this study. Figure 1a shows the genomic location of the amplicons in relation to the location of the *SERT* gene. Figure 1b shows sequences of amplicons 1 and 2. CpG units included in this study are numbered, underlined and highlighted in yellow. Primer sequences are highlighted in grey.

Supplementary Figure 1a.


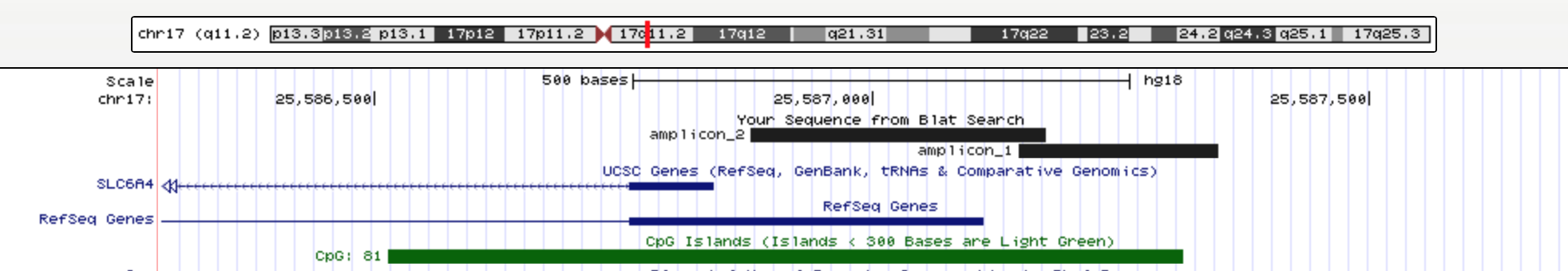


Supplementary Figure 1b.

Amplicon 1

CTGCTAGGTCCCAGGAAGAAAGAGAGAGCAGCTTTCGGGATGGGGACGATGGGGAGGTGTCCG

AGGTCAAGAGAAAGCGGCACGAGCAGACCCCTGTGTGCCGTCCTGTGGGCGCGGGGCGGCAG

GGGAGGCGCACACCTGCTCCTTTGTGCAGCCTCCCCCCTCC**CG**CAAAGTTAAAGAGCAGGAAA

*CpG unit*  1

GTCAGGATTCCT

Amplicon 2

TTAAAGAGCAGGAAAGTCAGGATTCCTCGCTCGGCCCTGCCCTGCCGGCTGCTCCGCGCTCCG

CTCCTCCCTGCGAGCGTGTGTGTGTGTCGGGGGTCCCTCCCCTCCTGGCTCTGGGGTCGGGCG

CGCACCC**CG**CCC**CG**TAG**CGCG**GCCCCTCCCTGG**CG**AGCGCAACCCCATCCAGCGGGAG**CGCG**

*CpG unit* 2 3 4

GAGC**CGCG**GC**CGCG**GGGAAGCATTAAGTTTATTCGCCTCAAAGTGACGCAAAAATTCTTCAAGA

5

GCTCTTTGG**CG**G**CG**GCTATCTAGAGATCAGACCATGTGAGGGCC

6
